# Influenza A and B Virus Attachment to Respiratory Tract in Marine Mammals

**DOI:** 10.3201/eid1805.111828

**Published:** 2012-05

**Authors:** Antonio J. Ramis, Debby van Riel, Marco W.G van de Bildt, Albert Osterhaus, Thijs Kuiken

**Affiliations:** Universitat Autònoma de Barcelona, Barcelona Spain (A.J Ramis);; Erasmus Medical Center, Rotterdam, the Netherlands (D. van Riel, M.W.G van de Bildt, A. Osterhaus, T. Kuiken)

**Keywords:** influenza, viruses, virus attachment, virus receptors, influenza A virus, influenza B virus, human influenza virus, avian influenza virus, marine mammals, respiratory tract

## Abstract

Patterns of virus attachment to the respiratory tract of 4 marine mammal species were determined for avian and human influenza viruses. Attachment of avian influenza A viruses (H4N5) and (H7N7) and human influenza B viruses to trachea and bronchi of harbor seals is consistent with reported influenza outbreaks in this species.

Understanding is limited about factors determining the ability of influenza viruses to cross the species barrier and persist in a new host population (*1**,**2*). In marine mammals, several subtypes of avian influenza A virus have caused epidemics in harbor seals (*Phoca vitulina*) (*3**–**6*). Also, human influenza B virus has been detected in harbor seals (*7*). These observations indicate the ability of both viruses to cross the species barrier and persist in harbor seals. In other marine mammal species, outbreaks of avian influenza A virus or infection with human influenza B virus have not been reported.

Attachment of influenza virus to tissues in the respiratory tract is a major determinant of host susceptibility to infection, efficiency of transmission, and pathogenicity and has been studied only to a limited degree (*8**,**9*). Attachment is determined largely by the specificity with which influenza virus attaches to sialosaccharide receptors on the host cell surface. In general, human influenza viruses prefer sialosaccharides in which sialic acid is linked to galactose by an α-2,3 linkage (SA-α-2,3-Gal), and avian influenza viruses prefer those with an α-2,6 linkage (SA-α-2,6-Gal) (*10*).

To understand differences in these properties between harbor seals and other marine mammals, we determined patterns of attachment for influenza virus strains known to have infected the respiratory tract of harbor seals, gray seals (*Halichoerus grypus*), harbor porpoises (*Phocoena phocoena*), and bottlenose dolphins (*Tursiops truncatus*). We chose gray seals, porpoises, and dolphins because their ranges overlap those of harbor seals and they are commonly kept in captivity.

## The Study

We determined patterns of attachment to respiratory tract tissues of 4 sympatric marine mammal species for several influenza viruses. Avian influenza A virus subtypes H7N7 (A/Seal/Massachusetts/1/80) and H4N5 (A/Seal/Ma/47/83) were chosen because they had caused outbreaks in harbor seals (*4**,**5*). An influenza B virus strain (B/Seal/Netherlands/1/99) was chosen because it had been isolated from a harbor seal (*7*).

For each of these 3 viruses, we also included a closely related strain from the putative donor host species (H7N7 A/Mallard/Sweden/100/02, H4N5 A/Mallard/Netherlands/13/2008, and B/Harbin/7/94, respectively) to determine whether adaptation to the new host species was associated with a change in attachment. Influenza virus A(H1N1)pdm09 (A/Netherlands/164/09) and seasonal subtype (H3N2) virus (A/Netherlands/213/03) were chosen because they circulate endemically in humans and might have contact with captive marine mammals through their caretakers. All viruses were isolated as described (*11**–**13*).

We obtained respiratory tract specimens from marine mammals from archives of paraffin-embedded tissues. Trachea and lung (including bronchus, bronchiole, and pulmonary alveoli) from 3 animals per species were examined.

Attachment of influenza virus to tissues was visualized by histochemical analysis as described (*13*). A positive result by light microscopy was granular to diffuse red staining on the apical surface of epithelial cells in trachea, bronchi, and bronchioles and on alveolar cells. Staining was scored as the percentage of cells in a section showing virus attachment. We also evaluated virus attachment to submucosal glands.

Results of attachment differed between avian influenza A viruses, human influenza A viruses, and human influenza B viruses. First, attachment of avian influenza A viruses to tracheal and bronchial epithelium was moderate in seals (harbor seal and gray seal) and absent in cetaceans (harbor porpoise and bottlenose dolphin) (Figure). Attachment to bronchiolar epithelium was moderate in seals and scarce in cetaceans, and attachment to alveolar epithelium was scarce in all 4 species. There were a few exceptions for virus attachment (Table). The source of avian influenza viruses (mallard or harbor seals) did not have a consistent effect on virus attachment in respiratory tract epithelium of any evaluated species (Table).

**Figure:**
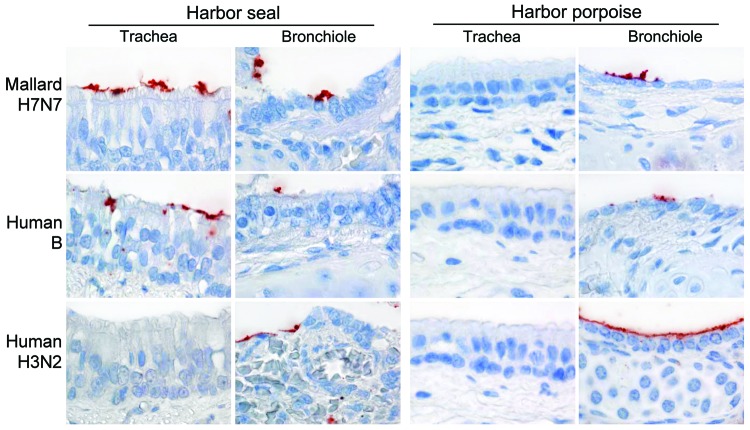
Attachment of 2 human influenza viruses and 1 avian influenza virus to trachea and bronchiole of harbor seal (*Phoca vitulina*) and harbor porpoise (*Phocoena phocoena*). Red staining indicates virus on the surface of epithelial cells (histochemical staining counterstained with hematoxylin; original magnification ×100).

**Table:** Attachment of mammal and human avian influenza viruses to respiratory tracts of 4 marine mammals*

Host (species) and virus strain	Level and cell tropism of virus attachment										
	Trachea			Bronchus			Bronchiole			Alveolus	
	Score	Predominant cell type		Score	Predominant cell type		Score	Predominant cell type		Score	Predominant cell type
Harbor seal (*Phoca vitulina*)											
Seal (H4N5)	+	Cil		+	Cil†		+	Cil		±	ND
Seal (H7N7)	+	Cil		+	Cil†		±	Cil		±	ND
Mallard (H4N5)	+	Cil		+	Cil†		+	Cil		±	ND
Mallard (H7N7)	+	Cil		+	Cil†		±	Cil		±	ND
Human (H1N1)	–			–			±	Cil		±	ND
Human (H3N2)	–			–			±	Cil		±	ND
Human B	+	Cil		+	Cil†		+	Cil		±	ND
Seal B	±	Cil		±	Cil†		±	Cil		±	ND
Gray seal (*Halichoerus grypus*)											
Seal (H4N5)	+	Cil†		+	Cil†		+	Cil		±	ND
Seal (H7N7)	+	Cil†		+	Cil†		+	Cil		±	ND
Mallard (H4N5)	+	Cil†		+	Cil†		+	Cil		±	ND
Mallard (H7N7)	+	Cil†		+	Cil†		+	Cil		±	ND
Human (H1N1)	–			–			–			–	
Human (H3N2)	–			–			–			–	
Human B	+	Cil†		+	Cil†		+	Cil		+	I
Seal B	±	Cil†		±	Cil†		±	Cil		±	ND
Harbor porpoise (*Phocoena phocoena*)											
Seal (H4N5)	±	Cil†		++	Cil		++	Cil		±	ND
Seal (H7N7)	–			–			±	Cil		±	ND
Mallard (H4N5)	–			–			±	Cil		±	ND
Mallard (H7N7)	–			–			±	Cil		±	ND
Human (H1N1)	–			±	Cil		+	Cil		++	I and II
Human (H3N2)	–			±	Cil		++	Cil		++	I and II
Human B	–			–			±	Cil		+	I
Seal B	–			–			±	Cil		+	I
Bottlenose dolphin (*Tursiops truncatus*)											
Seal (H4N5)	–			±	Cil		+	Cil		++	I and II
Seal (H7N7)	–			±	Cil		±	Cil		±	ND
Mallard (H4N5)	–			±	Cil		+	Cil		++	I and II
Mallard (H7N7)	±	Cil†		±	Cil		±	Cil		±	ND
Human (H1N1)	±	Cil†		±	Cil		+	Cil		+	I
Human (H3N2)	±	Cil†		±	Cil		++	Cil		++	I and II
Human B	–			–			±	Cil		+	I
Seal B	–			–			+	Cil		+	I

*Mean abundance of cells to which virus attached was scored as follows:+, moderate (10%–50% cells positive); ±, scarce (<10% cells positive); –, negative (no attachment); ++, abundant (>50% cells positive). Cil, ciliated epithelial cell; ND, not determined; I, type I pneumocytes; II, type II pneumocytes. †Virus attachment to submucosal glands.

Second, attachment of human influenza A viruses to tracheal and bronchial epithelium was absent in seals and scarce in cetaceans. Attachment to bronchiolar and alveolar epithelium was absent or scarce in seals and moderate to abundant in cetaceans (Table, Figure). We detected few differences between attachment of influenza virus A(H1N1)pdm09 and seasonal subtype (H3N2) virus to respiratory tract tissues of cetaceans (Table).

Third, attachment of influenza B viruses to respiratory tract epithelium at all levels was scarce to moderate in seals. Attachment was negative for tracheal and bronchial epithelium, scarce for bronchiolar epithelium, and moderate for alveolar epithelium in cetaceans (Table, Figure).

## Conclusions

Attachment of avian influenza A viruses to the respiratory tract was generally consistent with reports, or lack thereof, of avian influenza in these 4 marine mammal species. Moderate attachment of avian influenza A viruses to the trachea and bronchi of harbor seals suggests high susceptibility to and efficient transmission of these viruses. This finding is consistent with reported outbreaks of avian influenza in harbor seals (*4**–**6*). Scarce attachment of avian influenza viruses to bronchioles and alveoli of harbor seals is consistent with low pathogenicity of these viruses for harbor seals during experimental infection (*4**–**6*).

Attachment of avian influenza A virus to the respiratory tract in gray seals strongly resembles attachment in harbor seals. However, infection or outbreaks of avian influenza A virus in gray seals have not been reported, probably because virus attachment is required but is not sufficient for infection. Lack of attachment of avian influenza A viruses to trachea and bronchi of harbor porpoises and bottlenose dolphins suggests low susceptibility and inefficient transmission. This finding is consistent with lack of reported avian influenza A virus infections in these species (*14*).

Absence or scarcity of attachment of human influenza A viruses to trachea and bronchi of any of the marine mammal species contrasts with that of humans (*13*), in whom trachea and bronchi mainly express SA-α-2,6-Gal (*15*). This finding suggests low susceptibility to infection and can explain the lack of reported human influenza A virus infections in these 4 marine mammal species (*14*), even though they are often kept in captivity and are therefore at risk for infection from humans.

Attachment of influenza B virus to the respiratory tract of the 4 marine mammal species resembled that of the avian influenza A viruses. Moderate attachment of influenza B virus to the respiratory tract of seals suggests high susceptibility and efficient transmission. This finding is consistent with isolation of influenza B virus from a harbor seal and serologic evidence of influenza B virus infection in gray seals (*7*). Lack of attachment of influenza B virus to trachea and bronchi of cetaceans is consistent with absence of reported influenza B virus infections in these species.

Source of virus strain had little effect on its attachment. In general, there was high similarity of attachment of avian influenza A viruses from harbor seals and mallards. These findings suggest that avian influenza viruses do not require a different pattern of attachment to infect and transmit efficiently among harbor seals and that harbor seals might be susceptible to a wider range of avian influenza viruses than reported.

In conclusion, we report extensive diversity in the pattern of attachment of influenza viruses to the respiratory tract of marine mammals, which was determined by virus strain and host species involved. Our results correspond to field observations of influenza in marine mammals, i.e., outbreaks of avian influenza A virus and human influenza B virus infection in harbor seals (*4**,**5*) and lack of evidence of human influenza A virus infection in marine mammals. These results suggest that, as in humans (*11**,**15*), attachment of influenza virus to the proximal part of the respiratory tract, which depends largely on appropriate sialic acid moieties, is critical for susceptibility and efficient transmission of influenza viruses in marine mammals.
